# Anatomy of the dorsal default-mode network in conduct disorder: Association with callous-unemotional traits^☆^

**DOI:** 10.1016/j.dcn.2018.01.004

**Published:** 2018-01-28

**Authors:** Arjun Sethi, Sagari Sarkar, Flavio Dell’Acqua, Essi Viding, Marco Catani, Declan G.M. Murphy, Michael C. Craig

**Affiliations:** a Natbrainlab, Department of Forensic & Neurodevelopmental Sciences, Institute of Psychiatry, Psychology & Neuroscience, King’s College London, London, UK; b Sacker Institute for Translational Neurodevelopment, Department of Forensic & Neurodevelopmental Sciences, Institute of Psychiatry, Psychology & Neuroscience, King’s College London, London, UK; c Developmental Risk & Resilience Unit, University College London, London, UK; d Natbrainlab, Department of Neuroimaging, Institute of Psychiatry, Psychology & Neuroscience, King’s College London, London, UK; e National Autism Unit, Bethlem Royal Hospital, SLAM NHS Foundation Trust, Kent, UK

**Keywords:** Conduct disorder, Psychopathy, Default-mode network, Tractography, DTI, Diffusion MRI

## Abstract

We recently reported that emotional detachment in adult psychopathy was associated with structural abnormalities in the dorsal ‘default-mode’ network (DMN). However, it is unclear whether these differences are present in young people at risk of psychopathy. The most widely recognised group at risk for psychopathy are children/adolescents with conduct disorder (CD) and callous-unemotional (CU) traits. We therefore examined the microstructure of the dorsal DMN in 27 CD youths (14-with/13-without CU traits) compared to 16 typically developing controls using DTI tractography. Both CD groups had significantly (*p <* 0.025) reduced dorsal DMN radial diffusivity compared to controls. In those with diagnostically significant CU traits, exploratory analyses (uncorrected for multiple comparisons) suggested that radial diffusivity was negatively correlated with CU severity (Left: *rho* = −0.68, *p* = 0.015). These results suggest that CD youths have microstructural abnormalities in the same network as adults with psychopathy. Further, the association with childhood/adolescent measures of emotional detachment (CU traits) resembles the relationship between emotional detachment and network microstructure in adult psychopaths. However, these changes appear to occur in opposite directions – with increased myelination in adolescent CD but reduced integrity in adult psychopathy. Collectively, these findings suggest that developmental abnormalities in dorsal DMN may play a role in the emergence of psychopathy.

## 1 Introduction

Psychopathy is characterized by persistent antisocial behaviour and emotional detachment. The most commonly used instrument to measure these traits is the Psychopathy Checklist Revised (PCL-R), which quantifies emotional detachment (‘factor 1’) and antisocial behaviour (‘factor 2’) along separate dimensions (Hare, 2003). The underlying cause for psychopathy is most likely complex but there is compelling evidence that adults with psychopathy have differences in brain anatomy and function. For example we, and others, have previously reported that factors 1 and 2 are associated with microstructural abnormalities, consistent with reduced myelination, in a dorsal component of the default-mode network (DMN) (Sethi et al., 2015) and an amygdala–orbitofrontal limbic network (Craig et al., 2009; Motzkin et al., 2011; Passamonti et al., 2012) respectively. The dorsal DMN is of particular interest in the development of psychopathy due to the functions associated with it. Specifically, whilst the ventral component of the DMN (connecting the medial temporal lobe and posterior cingulate cortex (PCC)) is linked to autobiographical memory and spatial orientation (Catani and Thiebault de Schotten, 2012), the dorsal DMN, and the regions it connects (the medial prefrontal cortex and PCC), underpin affective (Kiehl et al., 2001; Maddock et al., 2003), social (Buckner et al., 2008; Völlm et al., 2006) and moral (Greene et al., 2001; Harrison et al., 2008) processing. In adult psychopathy, microstructural abnormalities within the dorsal DMN are linked to the affective and interpersonal differences that define the disorder (Sethi et al., 2015). However, the aetiology of these differences remains unclear.

Contemporary views suggest that adult psychopathy is the endpoint of a heritable neurodevelopmental disorder with its origins in early childhood (Frick and Viding, 2009). Indeed studies report that children with psychopathic traits display behavioural and neurocognitive differences that are similar to those found in adult psychopaths (Blair, 2013; Viding and McCrory, 2015). These psychopathic traits can be assessed using the Antisocial Process Screening Device (APSD; Frick and Hare, 2001), where emotional detachment is captured by the quantitative measurement of callous and unemotional (CU) traits. Importantly, interpersonal callousness and psychopathy measures in adolescence appear to predict adult psychopathy even when controlling for severity of adolescent antisocial behaviour (Burke et al., 2007; Lynam et al., 2007). Understanding whether dorsal DMN abnormalities previously described in adults with psychopathy are also related to CD and CU traits in childhood is therefore critical to characterising the developmental basis of the disorder.

Initial findings suggest this network may indeed be important for the development of psychopathy. Firstly, a recent twin study using the same tractography methods as our prior work (Sethi et al., 2015) revealed that the microstructure of the dorsal cingulum had moderate to high heritability, whilst the microstructure of the ventral cingulum was mostly determined by environmental factors (Budisavljevic et al., 2016). This indicates that the differences associated with this network appear to be heritable, and may reflect a basis for the heritability of psychopathy. This is further supported by findings that show that the grey matter of the PCC – which is one of the contributing nodes of the dorsal DMN – confers the heritability of CU traits (Rijsdijk et al., 2010). Secondly, recent studies have shown reduced functional connectivity within the DMN in CD (Broulidakis et al., 2016; Zhou et al., 2016). However, whether these changes reflect an underlying structural network deficit in those at risk of developing psychopathy has yet to be determined.

Therefore, in the current study we performed DTI tractography of the dorsal and ventral cingulum in 27 youths with conduct disorder (CD), 14 with and 13 without CU traits, as determined by DSM-V (Frick and Moffitt, 2010; Kahn et al., 2012), and 16 age and IQ matched typically developing controls. We hypothesised that, compared to controls, children with CD would have microstructural abnormalities in the DMN consistent with reduced myelination. Further, these abnormalities would be most severe in individuals with heightened levels of CU traits.

## 2 Methods

### 2.1 Participants

Twenty-seven right-handed male participants (aged 12–17) with CD were recruited from an Institute of Psychiatry database, Youth Offending Teams, Pupil Referral Units, youth projects and mainstream educational institutions as described in (Sarkar et al., 2013). These included 14 with, and 13 without, clinically significant CU traits. Sixteen right-handed controls from the same age range and inner city areas were also recruited through schools and youth services. Age, full scale IQ (FSIQ), ethnicity, substance use history, and ADHD diagnosis or hyperactive symptomatology did not differ significantly between groups, with two control, one CD-CU and one CD + CU youths having a prior diagnosis of ADHD. Aside from ADHD and CD, both cases and controls were medication free and neither group suffered from any other mental health problems. All participants fulfilled MRI safety criteria, spoke English as their first language, and had a FSIQ of 80–120. Participants gave full informed consent, with additional consent gained from parents/guardians when aged <16. This study was approved by the Joint South London and Maudsley Research Ethics Committee (243/00).

### 2.2 Questionnaires

Parent and self-report versions of the Strengths and Difficulties Questionnaire (SDQ; (Goodman, 1997)) were used to obtain measures of conduct problems and hyperactivity, and parent and self-report measures of the Antisocial Process Screening Device (APSD; (Frick and Hare, 2001)) were used to obtain measures of CU traits. Accepted subscales for both measures comprised the highest raters’ items (Jones et al., 2009; Sarkar et al., 2013). Postal versions of parental measures were used when parents did not accompany older participants. IQ was measured with the Wechsler Abbreviated Scale of Intelligence (WASI; (Wechsler, 1999)), and handedness by the Edinburgh Handedness Inventory (Oldfield, 1971).

### 2.3 Interviews and research diagnoses

Research diagnoses of CD were obtained using the CD and oppositional defiant disorder (ODD) subsections of the Kiddie Schedule for Affective Disorders and Schizophrenia Present and Lifetime Version (K-SADS-PL; (Kaufman et al., 1997)). Screening interviews were given to all participants, with those meeting CD or ODD criteria given complete interviews for both disorders. No participants met the criteria for ODD in the absence of CD. Interviews were conducted by a research psychologist (SS) and supervised by a psychiatrist (QD). Further information about antisocial behaviour was gained from teachers, social workers, parents and youth club workers. The CD group contained boys who had histories of serious, violent antisocial behaviour, including grievous bodily harm, sexual assault, robbery and burglary.

Research diagnosis of CD + CU was determined using proposed DSM specifier questions of the APSD (Frick and Moffitt, 2010; Kahn et al., 2012). These questions consist of four (lack of remorse or guilt, lack of empathy, unconcerned about performance, and shallow or deficient affect) of six questions from the callous-unemotional trait scale of the APSD. As recommended, answers of ‘Definitely True’ to at least two of these questions was taken as sufficient specification for the presence of callous-unemotional traits (Frick and Moffitt, 2010; Kahn et al., 2012).

### 2.4 Diffusion MRI acquisition

Diffusion MRI data were acquired using a GE Signa HDx 3.0T MR scanner (General Electric, USA), with actively shielded gradients (max amplitude 40 mT/m). The body coil was used for RF transmission, and eight-channel headcoil for signal reception, with a parallel imaging (Array Spatial Sensitivity Encoding Technique; ASSET) speed up factor of two. Head movement was minimised with extra padding, and a cardiac gated acquisition was used to minimise pulsatile cardiac artefacts in the parenchyma. Data were acquired using a multi-slice doubly refocused spin-echo echo planar imaging (EPI) sequence, optimised for parenchymal diffusion tensor measurement. Each volume consisted of 60 contiguous near-axial slices with a voxel size of 1.85 × 1.85 × 2.4 mm. TE was 104.5 ms with a TR varying between 12 and 20 RR intervals. Thirty-two diffusion weighted volumes were acquired with gradient directions distributed uniformly in space and a maximum B-value of 1,300 mm^2^/s, as well as four volumes with no diffusion weighting (Jones et al., 2002). The scanning sequence lasted approximately 15 min.

### 2.5 Diffusion MRI data processing

Data underwent extensive quality control checks, with all B0 and diffusion weighted volumes visually inspected for image corruption, motion artefacts and signal drop out. Datasets with more than two motion artefacts in different volumes on the same slice were removed from the study. Data showing head movements >1 cm were also excluded, though no data in this study met this criterion. Eddy current and motion correction was performed using ExploreDTI (Leemans et al., 2009), and the diffusion tensor estimated following removal of outlier data using RESTORE (Chang et al., 2005). Whole brain tractography was then performed with a step size of 0.5 mm using Euler integration with a b-spline interpolation of the tensor field (Basser et al., 2000), with an FA threshold of 0.2, and an angle threshold of 30°. Diffusion maps and tractography data were exported to TrackVis (Wang and Wedeen, 2007) for manual tract dissection.

### 2.6 Cingulum tractography

**Fig. 1:**
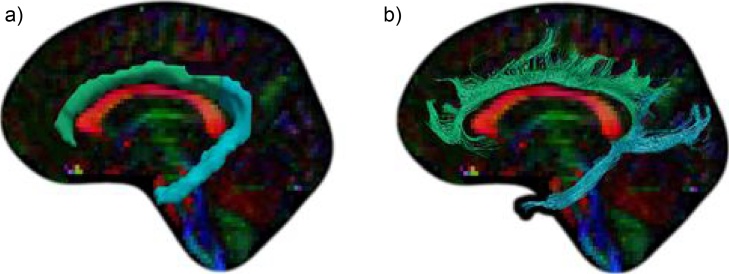
a) Regions of interest and b) Tractography of the dorsal (green) and ventral (blue) cingulum. (For interpretation of the references to colour in this figure legend, the reader is referred to the web version of this article.)

The cingulum was identified on the sagittal plane and defined using one region of interest (ROI), with an ROI at the midline to exclude callosal fibres. The ROI division between the dorsal and ventral cingulum was defined anatomically at the level of the splenium of the corpus callosum at the midline (Fig. 1), where an exclusion ROI was implemented to exclude fibres of the ventral cingulum that continued into the dorsal portion (Sethi et al., 2015). All dissections were performed blind to diagnosis. This methodology has been used to reliably show a) the different patterns of heritability in these two networks (Budisavljevic et al., 2016), and b) their differential contribution to psychopathy (Sethi et al., 2015).

### 2.7 Diffusion measures

Fractional anisotropy (FA) is derived from the three eigenvalues of the diffusion tensor and is a nonspecific but highly sensitive and widely used measure of microstructural architecture and white matter neuropathology (Alexander et al., 2007). Axial diffusivity represents the principal direction of diffusion within the tensor and has been shown to be more sensitive to axonal integrity and degeneration (↑ AD, ↑ axonal integrity). Radial diffusivity (RD) is a linear combination of the two eigenvalues perpendicular to the principal direction of diffusion and has been shown to be a more sensitive measure of myelination (↓ RD, ↑ myelination) (Alexander et al., 2007). Mean diffusivity (MD) offers a more general measure of the mean diffusion across the three eigenvalues, and is frequently used as a marker of oedema, inflammation and necrosis, but also the level of cellularity within a given voxel (Alexander et al., 2007). Here we used these four measures, to determine characterise differences in the microstructure of the dorsal and ventral cingulum between groups.

### 2.8 Statistics

General Linear Model statistics were applied to assess differences in FA, axial diffusivity (AD) and radial diffusivity (RD) in three separate models, with tract (dorsal and ventral cingulum) and hemisphere included as within subject factors, and group (control, CD-CU, CD + CU) as a between subjects factor whilst co-varying for age. As noted above, FA and MD are combined measures of the parallel (AD) and perpendicular (RD) components of the diffusion tensor. When outcome measures are not independent from one another, applying Bonferroni correction for each of them is overly conservative and prone to type II errors. We therefore employed an adjusted Bonferroni-corrected *p <* 0.025 for the two independent diffusion measures we used (AD and RD). We also report the effect of a more stringent Bonferroni correction of *p <* 0.0125 on results. Spearman’s *rho* partial correlations were used to assess the relationship of significantly different microstructural tract measures with CU traits whilst correcting for effects of age separately within each group. We report these post hoc exploratory correlational analyses uncorrected for multiple comparisons. We did not observe differences in age between groups (*p* = 0.959). However, due to the high variance in microstructure associated with age during this developmental period (Lebel et al., 2008), we corrected for age in all analyses to avoid differences associated with group or CU traits being masked by age-associated inter-individual differences in microstructure.

## 3 Results

### 3.1 Demographics

No significant differences in age or FSIQ were observed between groups (Table 1). As expected group differences were observed for conduct problems, CU traits, total APSD and total SDQ scores (Table 1). Importantly, pairwise comparisons revealed that both CD-CU (*p <* 0.001) and CD + CU (*p <* 0.001) groups significantly differed from controls on total conduct problems, but only the CD + CU group significantly different from controls in CU traits (*p* = 0.010). Additionally, both groups differed from controls in total SDQ scores (CD-CU: *p <* 0.001; CD + CU: *p* *=* 0.015), but only the CD + CU groups significantly differed from controls in total APSD scores (CD-CU: *p* = 0.309; CD + CU: *p* = 0.003). As previously reported (Sarkar et al., 2013), groups did not differ on previous substance use (*p >* 0.05).

**Table 1:** Demographics for controls and conduct disorder groups with and without callous unemotional traits.

Measure	Mean scores (SD)			F	DF	P
	Controls	CD-CU	CD + CU			
N	16	13	14			
Age	15.9 (1.83)	15.7 (1.80)	15.8 (2.11)	0.04	2, 40	0.959
FSIQ	103.6 (10.47)	98.3 (5.73)	99.2 (10.24)	1.44	2, 40	0.249
*SDQ Total*	11.8 (4.58)	17.0 (5.68)	19.4 (4.03)	9.67	2, 40	<0.001*
Conduct Problems	2.5 (1.36)	6.3 (2.52)	6.5 (1.90)	19.5	2, 40	<0.001*
Hyperactivity	6.4 (2.19)	6.4 (2.41)	7.8 (2.01)	1.65	2, 40	0.204
*APSD Total*	18.5 (6.21)	22.6 (7.12)	27.4 (6.71)	6.37	2, 40	0.004*
Callous unemotional traits	5.1 (2.33)	6.4 (2.13)	7.8 (2.31)	4.89	2, 40	0.013*

CD-CU = Children with Conduct Disorder but without clinically significant Callous Unemotional Traits; CD + CU = Children with Conduct Disorder and with clinically significant Callous Unemotional Traits; SDQ = Strengths and Difficulties Questionnaire; APSD = Antisocial Process Screening Device.*Significant at p < 0.05

### 3.2 Group differences in microstructure

A significant between factors effect for group was observed (*F*(1,39) = 10.38, *p* = 0.008), with radial diffusivity in the bilateral dorsal and left ventral cingulum significantly differing between groups (Table 2). *Post hoc* pairwise comparisons revealed significantly reduced RD in the dorsal cingulum between controls and CD + CU (Left: *p* = 0.021; Right: *p* = 0.005) and CD-CU (Left: *p* = 0.008; Right: *p* = 0.002) groups. No effect of group was observed in the ventral cingulum (Left: *p* = 0.112; Right: *p* = 0.032) after accounting for multiple comparisons (*p <* 0.025). If we use a more conservative Bonferroni correction (*p <* 0.0125) then all previously significant results survive except the difference between CD + CU and controls in the left hemisphere (*p* = 0.021). No significant differences were observed between the CD + CU and CD-CU groups in pairwise comparisons. No significant differences in FA (*p* = 0.039), AD (*p* = 0.250) or MD (*p* = 0.091) between groups were observed after correction for multiple comparisons (*p <* 0.025).

**Table 2:** Between group differences in cingulum subnetwork diffusion measurements.

Measure	Mean scores (SD)			F	DF	p
	Controls	CD-CU	CD + CU			
**Radial Diffusivity**	*Omnibus test for group effects:*			5.55	1, 39	0.008*
*Left*						
Dorsal Cingulum	0.597 (0.0287)	0.572 (0.0308)	0.574 (0.0244)	4.65	1, 39	0.015*
Ventral Cingulum	0.613 (0.0411)	0.590 (0.0296)	0.601 (0.0251)	2.32	1, 39	0.112
*Right*						
Dorsal Cingulum	0.603 (0.0242)	0.574 (0.0303)	0.576 (0.0257)	6.97	1, 39	0.003*
Ventral Cingulum	0.597 (0.0316)	0.571 (0.0279)	0.584 (0.0264)	3.76	1, 39	0.032

**Fractional anisotropy**	*Omnibus test for group effects:*			3.54	1, 39	0.039
*Left*						
Dorsal Cingulum	0.456 (0.0200)	0.462 (0.0216)	0.464 (0.0187)	–	–	–
Ventral Cingulum	0.407 (0.0228)	0.417 (0.0191)	0.402 (0.0215)	–	–	–
*Right*						
Dorsal Cingulum	0.434 (0.0214)	0.448 (0.0262)	0.448 (0.0185)	–	–	–
Ventral Cingulum	0.387 (0.1054)	0.426 (0.0139)	0.416 (0.0188)	–	–	–

**Axial diffusivity**	*Omnibus test for group effects:*			1.44	1, 39	0.250
*Left*						
Dorsal Cingulum	1.253 (0.1293)	1.250 (0.0319)	1.265 (0.0330)	–	–	–
Ventral Cingulum	1.187 (0.0449)	1.164 (0.0339)	1.151 (0.0331)	–	–	–
*Right*						
Dorsal Cingulum	1.233 (0.0481)	1.205 (0.0226)	1.213 (0.0459)	–	–	–
Ventral Cingulum	1.171 (0.0412)	1.146 (0.0267)	1.152 (0.0370)	–	–	–
**Mean diffusivity**	*Omnibus test for group effects:*			2.55	2, 39	0.091
*Left*						
Dorsal Cingulum	0.828 (0.0306)	0.746 (0.0193)	0.804 (0.0255)	–	–	–
Ventral Cingulum	0.804 (0.0419)	0.763 (0.0699)	0.784 (0.0509)	–	–	–
*Right*						
Dorsal Cingulum	0.812 (0.0239)	0.731 (0.0199)	0.788 (0.0278)	–	–	–
Ventral Cingulum	0.788 (0.0318)	0.751 (0.0456)	0.773 (0.0262)	–	–	–

CD-CU = Children with Conduct Disorder but without clinically significant Callous Unemotional Traits; CD + CU = Children with Conduct Disorder and with clinically significant Callous Unemotional Traits.

* *Significant at p <* *0.025*.

### 3.3 Relationship between CU traits and microstructure

**Fig. 2:**
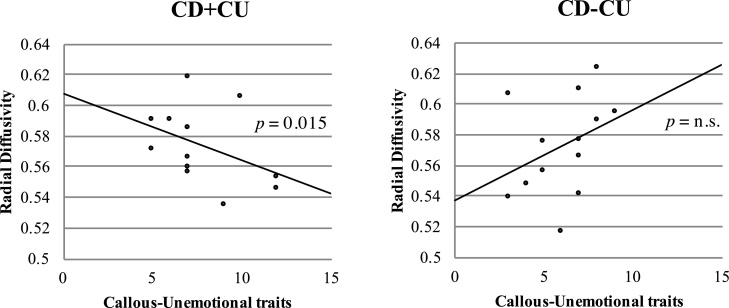
Relationship between radial diffusivity and CU traits in CD + CU (*rho* = −0.68, *p* = 0.015) (and CD-CU *rho* = 0.39, *p* = 0.184) in the left dorsal cingulum after correcting for age.

As we observed no evidence of between group differences between the CD-CU and CD + CU groups, we performed exploratory post hoc analyses to assess if differences within either clinical group separately may be associated with CU traits. Greater CU traits were negatively correlated with radial diffusivity in the left dorsal cingulum in CD youths with, but not without, CU traits (*rho* = −0.68, *p* = 0.015; *rho* = 0.39, *p* = 0.184 respectively, Fig. 2). CU traits were not significantly correlated with radial diffusivity in the right dorsal cingulum (CD + CU: *rho* = −0.326, *p* = 0.301; CD-CU: *rho* = 0.342, *p* = 0.253), or the left (CD + CU: *rho* = −0.379, *p* = 0.224; CD-CU: *rho* = 0.213, *p* = 0.484) or right (CD + CU: *rho* = −0.018, *p* = 0.957; CD-CU: *rho* = 0.185, *p* = 0.546) ventral cingulum.

## 4 Discussion

We report that CD in male adolescents is associated with reduced radial diffusivity (RD) of the central component of the dorsal default-mode network (DMN) – the dorsal cingulum. This is important as the regions that the dorsal DMN subsumes are central to affective (Kiehl et al., 2001; Maddock et al., 2003), social (Buckner et al., 2008; Völlm et al., 2006) and moral (Greene et al., 2001; Harrison et al., 2008) processing. These changes are suggestive of increased myelination in this network (Alexander et al., 2007). This contrasts with studies in adults with psychopathy, which report changes consistent with reduced myelination in this network (Sethi et al., 2015).

In children with CD, ‘emotional detachment’ is captured within the construct of CU traits. The presence of these traits in adolescence is associated with increased risk of developing psychopathy in adulthood (Burke et al., 2007; Lynam et al., 2007). Therefore, we completed a between group analysis of dorsal DMN RD (CD + CU vs. CD-CU), and also assessed continuous relationships between CU traits and dorsal DMN RD in each group. The dorsal DMN RD did not differ when comparing the CD + CU and CD-CU groups. However, in post hoc and Bonferroni-uncorrected exploratory analyses we found a significant correlation between CU traits and RD in the CD + CU group only. This suggests that there might be aetiologically distinct pathways to reduced dorsal DMN RD in the two CD groups. Specifically, the pattern appears to be positively correlated to CU traits in the CD + CU group, but associated with some other factor in the CD-CU group. However, more statistically rigorous replication of this finding in a larger sample and a richer measure of CU traits (e.g. the Inventory of Callous-Unemotional Traits (Kimonis et al., 2008)) is needed.

Another significant difference between our study of children with CD compared to adults with psychopathy is the direction of change in the microstructural integrity of the dorsal DMN. Our proxy measure of myelin content (RD) suggests that, compared to controls, CD is associated with *increased* myelination (Song et al., 2002), whereas earlier findings in adult psychopathy found the opposite (Sethi et al., 2015). This inverse pattern of proxy measures of myelination can also been found when comparing studies of the uncinate fasciculus (UF) in CD children/adolescents (Passamonti et al., 2012; Sarkar et al., 2013; Zhang et al., 2014) and adult psychopaths (Craig et al., 2009; Motzkin et al., 2011; Passamonti et al., 2012).

One explanation for these findings is that antisocial behaviour is associated with differences in the neurodevelopmental trajectory of specific white matter tracts (e.g. UF and dorsal DMN), More specifically, there may be an initial *acceleration* of myelination during childhood/early adolescence followed by a relative *deceleration* in adulthood (i.e. a *‘Hare and Tortoise’* effect (Aesop, 1867)). Although highly speculative, a recent longitudinal study in children with autism spectrum disorder (ASD), lends some support to this suggestion (Wolff et al., 2012). This longitudinal analysis reported that, compared to controls, there was an initial increase in fractional anisotropy in specific tracts in children with ASD, followed by a blunted developmental trajectory. While these findings were reported in earlier childhood, it is noteworthy that the UF and dorsal DMN are among the last to fully mature in the human brain (Lebel et al., 2008). It was not possible to address this developmental hypothesis in the present sample due to its small size and limited age range. Future work specifically powered to address this question within a large cross-sectional or, ideally, longitudinal sample will be necessary to address this hypothesis directly.

It will also be important for future work to directly test the relationship between measures of ‘structural’ and ‘functional’ connectivity in CD. Some prior studies examining functional connectivity in adolescents with CD have, for example, reported reduced connectivity in the DMN (Broulidakis et al., 2016; Zhou et al., 2016). It remains unclear whether this apparent discrepancy is due to differences in sampling strategies or other methodological factors.

In addition to the need for longitudinal studies to test the above hypothesis in antisocial behaviour, similar studies are also needed to determine whether this trajectory is fixed or reversible. It has been reported, for example, that on completion of a group parenting programme to treat CD (+/−CU), that up to 80% of children no longer fulfil criteria for CD and this figure remains at 65% at 6-month follow-up (Furlong et al., 2013; Hawes and Dadds, 2005; Webster-Stratton and Hammond, 1997). Further, this is still significant 6–10 years later (Scott et al., 2014). However, to date nobody has examined the relationship between these behavioural changes and changes in the neurodevelopmental trajectory of these specific tracts. It is hoped that such future studies will enable us to better understand the biological basis of antisocial behaviour and psychopathy and ultimately to develop more effective treatments at key points along the neurodevelopmental trajectory.

## Conflict of interest

None.
